# Belief inflexibility reduces the impact of allostatic overload on psychotic-like experiences among Ukrainian refugees

**DOI:** 10.3389/fpsyt.2026.1761027

**Published:** 2026-03-17

**Authors:** Julian Maciaszek, Julia Aleinikova, Agnieszka Dybek, Błażej Misiak

**Affiliations:** Department of Psychiatry, Wroclaw Medical University, Wroclaw, Poland

**Keywords:** allostatic load, belief inflexibility, psychotic-like experiences, refugees, war trauma

## Abstract

**Introduction:**

Refugees are chronically exposed to cumulative stress, which may increase allostatic load (AL) and vulnerability to psychotic-like experiences (PLEs). Belief inflexibility, reflecting difficulties in revising beliefs in response to new information, may influence how physiological stress affects psychosis proneness. This study examined whether AL index interacts with cognitive biases and refugee status in predicting PLEs.

**Methods:**

Sixty Ukrainian refugees and fifty matched controls underwent psychiatric evaluation, assessment of cognitive biases, and measurement of AL biomarkers.

**Results:**

Refugees had fewer years of education and showed higher depressive, anxiety, PLEs, trauma-related symptoms, greater belief inflexibility and elevated AL index. Significant positive correlations were found between AL index and PLEs and between AL index and belief inflexibility. Moderation analyses revealed that both AL index and belief inflexibility were positively associated with PLEs, and refugee status amplified these effects. However, the interaction between AL index and belief inflexibility was negative, suggesting that higher cognitive rigidity weakened the direct impact of physiological stress on PLEs. Subsequent analyses indicated that this attenuating effect was especially pronounced in the refugee group.

**Discussion:**

These findings highlight the complex interplay between stress physiology andcognitive style in shaping psychosis vulnerability and adaptation in trauma exposed refugee populations.

## 1 Introduction

The large scale Russian invasion of Ukraine in early 2022 forced nearly seven million people to flee the country, with approximately 3.5 million mainly women and children seeking refuge in Poland (1). Refugees have been exposed to war related trauma and migration related stress, including family separation, uncertainty, and adaptation challenges. At the same time, Poland provided substantial humanitarian and institutional support, ensuring access to healthcare, education, and legal protection. Against this background of cumulative and prolonged stress exposure, objective indicators of physiological burden become particularly relevant. The allostatic load (AL) index is a measure used to assess the physiological impact of prolonged exposure to stress on the body (2). Indeed, the AL index, which encompasses markers from the endocrine, immune-inflammatory, metabolic, and cardiovascular systems, is an effective predictor of adverse stress-related outcomes (3). Research involving individuals with psychiatric disorders, such as mood and psychotic conditions, have demonstrated that the AL model is a useful framework for examining the biological effects of psychosocial stress (4–7). AL index is also applicable in general population as it provides insights into the cumulative physiological impacts of stress and can predict health outcomes across diverse demographic groups (8). While it is well-established that stress from disasters like war-related migration affects various biological systems, there has been limited research on using objective physiological stress measures, such as AL index, in relation to disaster-related outcomes, including refugees (9). One systematic review and meta-analysis have demonstrated that Refugees represent a population at particularly high risk for psychosis-related outcomes, as substantially increased rates of non-affective psychotic disorders among refugees compared with both native populations and non-refugee migrants (10). Corresponding findings were found in the study of Bosnian war refugees with post-traumatic stress disorder (PTSD) and depression found a blunted cortisol awakening response and lower daytime cortisol compared to healthy controls (11). In line with these considerations, another research examining Somali refugees found that exposure to trauma and the perceived stress level were associated with lower levels of cortisol, which can indicate a blunted stress response, often linked to chronic stress or repeated trauma (12). Another study in contrary found no direct link between post-displacement stress and cortisol or dehydroepiandrosterone sulfate (DHEA-S) levels (13). These inconsistencies may reflect differences in the chronicity and phase of stress exposure, as acute stress is typically associated with hyperactivation of the hypothalamic–pituitary–adrenal (HPA) axis, whereas prolonged or repeated trauma may lead to hypocortisolism and a blunted stress response due to allostatic overload. Moreover, heterogeneity in sampling procedures, timing of cortisol assessment, and post-migration environmental factors may further contribute to divergent findings across refugee studies. Importantly, the AL framework provides a biologically grounded link between chronic stress exposure and stress-sensitive psychiatric phenotypes, including presence of. psychotic-like experiences (PLEs). PLEs refer to transient or mild symptoms that resemble psychosis, such as hallucinations, delusions, or disorganized thinking, often occurring in non-clinical populations (14). PLEs are considered particularly sensitive to sustained dysregulation of stress-related systems, including the hypothalamic-pituitary-adrenal axis and immune-inflammatory pathways captured by the AL index (15). Therefore, examining AL in relation to PLEs allows for an integrated investigation of how cumulative physiological stress translates into subclinical psychosis vulnerability, especially in trauma-exposed refugee populations.

It was shown that psychosocial stress has been shown to elevate the risk of experiencing psychotic-like symptoms, making refugees a high-risk group for psychosis (14). Similarly, one study found that refugees and asylum seekers were at a significantly higher risk of experiencing psychotic symptoms compared to the general population (16). This increased risk has been associated with the history of trauma, perceived stress levels, and socioeconomic challenges faced by these populations. Additionally, another study reported that refugees had higher rates of PLEs compared to non-refugee populations (17). Similarly to previous studies, it was found that Afghan migrant students in Iran reported a higher prevalence of PLEs compared to non-migrant peers (18). Given that PLEs are sensitive to cumulative stress exposure, identifying factors that may moderate the relationship between allostatic load and PLEs is a critical step toward understanding individual differences in stress-related vulnerability. Chronic high stress weakens flexible, hippocampal–prefrontal cognitive processes while relatively preserving habitual, striatum and amygdala-based processing, providing a potential pathway through which physiological stress burden may influence the expression of PLEs. In this context the elevated vulnerability to PLEs among refugees has been linked not only to stress exposure but also to cognitive biases which are systematic patterns of deviation from norm or rationality in judgment, leading individuals to process information in ways that can result in inaccurate conclusions or irrational decisions (18). Cognitive biases were shown to be positively associated with the onset and persistence of hallucinations and delusions among refugees (19). One study highlighted that certain biases, including jumping to the conclusions were more prevalent in populations with the history of trauma, including refugees (20). Moreover, a meta-analysis showed that jumping to conclusions and belief inflexibility biases were consistently more pronounced in individuals exposed to severe stress or displacement (21). It has been also reported that individuals who had higher levels of selective attention for threat bias reported more PLEs (22). Furthermore, the study revealed that the level of stress mediated the relationship from cognitive biases to PLEs (22).

Although cognitive rigidity is typically conceptualized as a vulnerability factor for psychosis (23–28), accumulating evidence suggests that related cognitive biases may confer short-term psychological benefits. External attribution bias has been shown to attenuate associations between anxiety and positive PLEs, while attention-to-threat bias weakens links between depressive symptoms and PLEs (29). At the same time, meta-analytic findings indicate that individuals with PLEs exhibit stronger jumping-to-conclusions and externalizing attribution biases, which are positively associated with PLE severity (30), highlighting the dual role of rigid cognitive styles as both protective and costly. In line with this dual-role framework, constructs closely related to belief inflexibility, such as need for closure and decisiveness, have been associated with reduced uncertainty and anxiety in ambiguous environments (31, 32), and previous work from our group demonstrated a negative association between decisiveness and PLEs in a large cross-population sample (33). Within the framework of the present study, belief inflexibility emerges in our opinion as a particularly relevant cognitive bias, reflecting a reduced tendency to revise interpretations or beliefs in response to disconfirming evidence and operationalized as the bias against disconfirmatory evidence (34). Empirical studies have consistently shown that the bias against disconfirmatory evidence is elevated in individuals with schizophrenia and is also present in non-clinical populations with higher delusion proneness, supporting its role across the psychosis continuum (35).

Meta-analytic evidence further indicates that impaired integration of disconfirming evidence is robustly associated with delusions and delusion severity, highlighting belief inflexibility as a core reasoning abnormality relevant to psychosis-related phenotypes (36.

PLEs in the general population have similarly been linked to alterations in belief updating and reasoning biases, suggesting that rigid belief maintenance may contribute to subclinical psychosis expression (37).

Given that forced displacement is associated with prolonged exposure to cumulative stressors and elevated AL, belief inflexibility in our opinion may constitute a key cognitive mechanism moderating the relationship between physiological stress burden and the emergence of PLEs (15).

To the best of our knowledge, previous studies have not explored the relationships between AL index, psychopathological symptoms and cognitive biases in refugee populations. Consequently, this study has two primary objectives: first, to compare the AL index between refugees and a control group, and second, to evaluate whether cognitive biases moderate the association between the AL index and PLEs.

## 2 Material and methods

### 2.1 Participants

The study involved 60 Ukrainian refugees who crossed the Polish-Ukrainian border following the Russian invasion on February 24, 2022 (11 males and 49 females, with an average age of 27.8 ± 6.1 years), alongside 50 healthy control participants (14 males and 36 females, with an average age of 26.1 ± 3.4 years). Participants were recruited through advertisements shared on social media and the websites of the Department of Psychiatry at Wroclaw Medical University and the Polish Psychiatric Association. The study protocol received approval from the Bioethics Committee at Wroclaw Medical University (approval number: KB - 97/2023), and all participants provided written informed consent. Both refugees and controls underwent psychiatric evaluations during the study period of 2022–2023 using the Mini-International Neuropsychiatric Interview (38), a structured diagnostic interview designed to assess major DSM-5 and ICD-10 psychiatric disorders. Psychiatric and psychological assessments were conducted by trained psychiatrists and clinical psychologists. Assessors working with Ukrainian participants were fluent in Ukrainian or Russian, ensuring accurate communication. Participants were interviewed in a quiet clinical setting at the Department of Psychiatry, Wroclaw Medical University, prior to MRI scanning, which was performed on the same day or within a short time interval. Exclusion criteria for both groups included (1): age under 18 or over 65; (2) concurrent neurological disorders; (3) cognitive impairment; (4) physical health conditions that might affect the biochemical markers evaluated (including diabetes, hypertension, coronary artery disease, autoimmune disorders, inflammatory diseases, and endocrine issues); (5) substance and/or alcohol dependency (excluding nicotine); and (6) specific psychiatric diagnoses as per ICD-10 criteria, such as organic mental disorders (F00 - F09), schizophrenia-spectrum disorders (F20-F29), type I bipolar disorder (F31), and severe personality disorders (F60-F69). Refugees and control participants were matched based on age and sex. To minimize potential language barriers, informed consent and study procedures were conducted with the assistance of a team member fluent in Ukrainian and Russian when required, and all questionnaires were administered using standardized Ukrainian and or Russian versions.

### 2.2 Measures

#### 2.2.1 The Prodromal Questionnaire-16

The PQ-16 has been developed to screen for psychosis risk states (39). It includes 16 items with two corresponding subscales. The first subscale refers to the presence of specific psychotic experiences (yes-or-no responses) while the second one measures associated distress (4-point responses, where 1 means lack of distress and 4 means severe distress). The Cronbach’s alphas in the present study was 0.878 for the distress subscale and 0.831 for the presence subscale.

#### 2.2.2 The Davos Assessment of the Cognitive Biases Scale

The DACOBS is a self-report questionnaire developed to measure a variety of cognitive biases associated with psychotic disorders including jumping to conclusions, belief inflexibility, selective attention for threat, external attribution bias, social cognition problems, subjective cognitive problems, and safety behaviors (40). It consists of 42 questions, with responses rated on a 7-point scale (where 7 means “I definitely agree” and 1 means “I definitely disagree”. The Cronbach’s alpha of the DACOBS was 0.878 in the present study.

#### 2.2.3 The General Anxiety Disorder-7

The GAD-7 is a tool used to assess the severity of generalized anxiety symptoms (41). It includes seven self-reported items, each rated on a scale from 0 (“not at all”) to 3 (“nearly every day”), yielding a total score between 0 and 21. Higher scores reflect greater levels of generalized anxiety symptoms. In the current study, the GAD-7 demonstrated strong reliability, with a Cronbach's alpha of 0.87.

#### 2.2.4 The Beck Depression Inventory

The BDI is a 21-item self-assessment measure designed to evaluate typical attitudes and symptoms associated with depression (42). Items are rated from 0 to 3 points, where 0 refers to a lack of depressive symptoms (e.g., “I am not sad or depressed”) and 3 corresponds with severe depressive symptoms (e.g., “I am constantly sad or unhappy, its unbearable”). Total score is ranging from 0 to 63, with higher scores indicating more severe depressive symptoms. In this study, the BDI showed excellent reliability, with a Cronbach's alpha of 0.89.

#### 2.2.5 The Impact of Event Scale-Revised

The IES-R is a 22-item questionnaire used to identify PTSD-related symptoms (43). It is divided into three subscales, representing groups of symptoms related to PTSD: intrusion, hyperarousal, and avoidance. Answers to each item are given using a five-point Likert scale, rated from 0 (“not at all”) to 4 (“definitely yes”). The total IES-R score ranges between 0 and 88 (higher scores indicate higher levels of PTSD symptoms). The Cronbach’s alpha in the current study was 0.93 for IES-R total score; 0.91 for intrusion subscale; 0.87 for hyperarousal subscale and 0.93 for avoidance subscale.

### 2.3 Biochemical measurements

Two venous blood samples were obtained from participants between 7 and 9 a.m. after they had fasted overnight. One sample underwent centrifugation at 2500 rpm for 10 minutes to separate the serum, which was subsequently stored in aliquots at −80 °C. The second sample was used for measuring fibrinogen levels. Colorimetric methods were applied using the Konelab 60 analyzer (Argenta) to determine glucose, albumin, total cholesterol, high-density lipoproteins (HDL), and triglycerides. Glycated hemoglobin (HbA1c) levels were assessed through high-performance liquid chromatography, while low-density lipoproteins (LDL) were calculated using the Friedewald equation (44). The levels of cortisol, insulin, and dehydroepiandrosterone sulfate (DHEA-S) were measured using electrochemiluminescence analysis with the Cobas e411 analyzer (Roche). High-sensitivity C-reactive protein (hsCRP) levels were assessed using the immunonephelometric method with the BN2 analyzer (Siemens), and interleukin-6 (IL-6) levels were determined via the sandwich enzyme immunoassay technique (R&D Systems Inc., MN, USA). Finally, fibrinogen concentrations were measured using the coagulometric Clauss method along with the QFA Thrombin Reagent (Werfen IL) and the ACL TOP500 analyzer.

### 2.4 Allostatic load

The AL index was calculated using 15 parameters grouped into six distinct categories of markers: 1) anthropometric measures, including body mass index (BMI) and waist-to-hip ratio (WHR); 2) cardiovascular indicators such as resting heart rate, systolic blood pressure, and diastolic blood pressure; 3) lipid profile markers, comprising total cholesterol, LDL, HDL, and triglycerides; 4) glucose homeostasis factors, including glucose, HbA1c, and insulin; 5) neuroendocrine markers represented by cortisol and DHEA-S; and 6) immune-inflammatory indicators, which include hsCRP, fibrinogen, albumin, and IL-6. These markers were chosen based on recent tools designed to assess physiological dysregulations and comorbidities in individuals with severe mental health conditions (45). While albumin levels are typically viewed as indicators of renal function and nutritional status, prior studies on the AL index in various populations have also considered albumin as a marker of inflammation. The AL index was calculated using the percentile distribution of the 15 biomarkers among control participants, following a widely accepted methodology (46). Specifically, the 75th percentile (and the 25th percentile for HDL and albumin) was determined based on the distribution within the control group, with separate cutoff percentiles established for males and females. The total number of markers exceeding the 75th percentile (and those falling below the 25th percentile for DHEA-S, HDL, and albumin) in each category was divided by the total number of markers in that category to ensure an equal contribution from each biological system to the AL index. The scores from all categories were then summed to compute the final AL index.

### 2.5 Statistical analysis

Differences in the distribution of categorical variables were tested using the χ2 test. The Kolmogorov-Smirnov test was used to assess the distribution of continuous variables. Differences in continuous variables between refugees and controls were analyzed using the t-tests (in case of data with normal distribution) or the Mann-Whitney U test (in case of data with non-normal distribution). Correlations between continuous variables were tested using the Spearman’s rank coefficients. The analysis of co-variance (ANCOVA) was used to test the effect of group (refugees vs. controls) on the AL index after co-varying for the age, sex, presence of chronic somatic diseases and number of years with completed education. Moderation was tested in the PROCESS macro (Model 3) (47). Additionally, a sensitivity power analysis conducted in G*Power (F test; linear multiple regression, fixed model, R² increase) indicated that with N = 110, α = 0.05, and 80% power, the study was sufficiently powered to detect small-to-moderate incremental interaction effects in the final moderation model (f² = 0.082–0.128; df_2_ = 98) (48).The AL index was included as a predictor (X variable), while PLEs (PQ-16, presence subscale) was included as the outcome variable (Y variables). The cognitive biases were included as a first moderator (moderator variable W) while refugee status was included as a second moderator (moderator variable Z). Age, sex, the number of years with completed education and somatic disease status were added as covariates. The Johnson-Neyman technique was used to identify the range of belief inflexibility bias for which the interaction effect is significant. Results were considered as significant after adjustment for multiple testing using the Benjamini–Hochberg procedure (p ≤ 0.006).

## 3 Results

**Figure 1:**
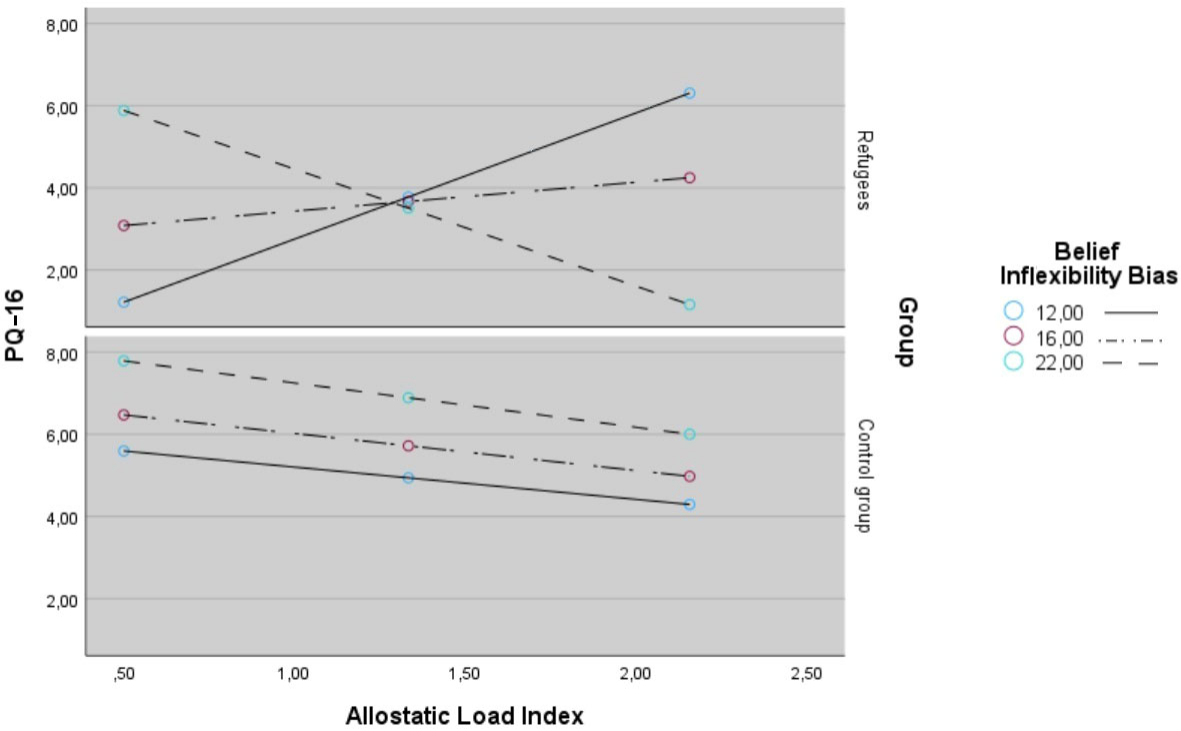
The allostatic load index in refugees and controls (error bars represent 95%CI, p < 0.001).

**Figure 2:**
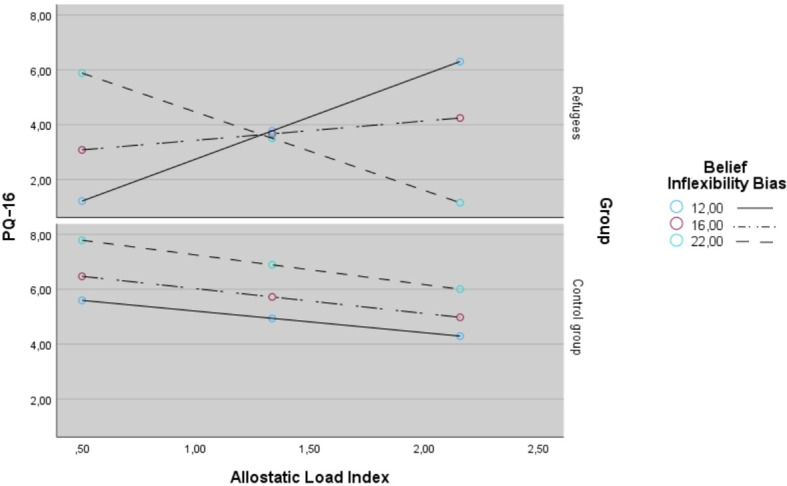
Conditional effects of the focal predictor (scores of allostatic load index) at values of the moderator (belief inflexibility bias) in both groups. *PQ-16 - the Prodromal Questionnaire-16. AL – allostatic load*.

The general characteristics of refugees and controls are presented in **Table 1**. There were no significant differences between both groups in terms of socio-demographic characteristics (age, sex, somatic disease and cigarette smoking status) except for the number of years of completed education that was significantly lower in refugees (17.3 ± 2.3 vs. 13.7 ± 2.9; p < 0.001). Refugees, in comparison with healthy controls, had significantly higher scores in both presence (5.8 ± 3.6 vs. 3.3 ± 3.1; p < 0.001) and distress subscales of PQ-16 (11.6 ± 9.7 vs. 6.8 ± 6.7; p = 0.005), BDI (13.3 ± 7.6 vs. 7.9 ± 7.8; p < 0.001), GAD-7 (8.5 ± 5.0 vs. 6.0 ± 4.7; p = 0.006) as well as in avoidance (10.1 ± 5.2 vs. 7.3 ± 4.9; p = 0.005)and hyperarousal subscales of IES-R. Refugees showed also significantly higher level of belief inflexibility (18.1 ± 5.6 vs. 15.1 ± 3.9; p = 0.001). The AL index was significantly higher in refugees compared to controls (**Figure 1**, 1.6 ± 0.7 vs. 1.1 ± 0.7, p < 0.001). This difference remained significant (F = 4.81, p = 0.032) after co-varying for age (F = 5.21, p = 0.03), sex (F = 0.86, p = 0.356), presence of chronic somatic diseases ( (F = 0.22, p = 0.644), and the number of education years (F = 1.73, p = 0.192). Bivariate analyses of individual AL index components (**Table 2**) revealed significantly lower levels of cortisol in refugees compared to controls, which remained significant after Benjamini–Hochberg correction for multiple comparisons (p **<** 0.001). Correlations of the AL index with psychotic-like experiences, psychopathological symptoms and cognitive biases are shown in **Table 3**. After adjustment for multiple testing, there were found the significant positive correlations between the AL index and the score of the presence subscale of PQ-16 (r = 0.186, p = 0.016) and between AL index and the level of belief inflexibility (r = 0.251, p = 0.010). Results of moderation analysis are reported in **Table 4**. There was significant positive association of AL index with PLEs (B = 10.179 ± 3.830, p = 0.009) and there was also significant positive association of belief inflexibility with PLEs (B = 0.763 ± 0.265, p = 0.005). Additionally it was found a positive association of refugee status and PLEs and there was positive association of AL index × refugee status and PLEs. There was significant negative association of AL index × belief inflexibility and PLEs (B = −0.592 ± 0.253, p = 0.021). As well there was also significant negative association of AL index × belief inflexibility × refugee status with PLEs (B = −0.563 ± 0.272, p = 0.018). The model explained 30.2% of variance in the presence subscale of PQ-16 (*R^2^* = 0.302). Adding the interaction term increased the *R^2^* value in the refugees model. Adding the interaction term to the refugee group model was associated with a significant *R^2^* change (*R^2^* change = 0.086, *F*  = 5.228, *p*  = 0.021). The PQ-16, presence score defining the Johnson-Neyman region of significance in refugee group model was 15.280 (% of non-significant correlations below the cut-off = 56.000 and % of significant correlations above the cut-off = 44.000). Conditional effects of the focal predictor (scores of AL index) at values of the moderator (belief inflexibility bias) in both groups are shown in **Figure 2**. Regression lines were plotted for the following levels of belief inflexibility bias: (1) low level = 12.0 (control group: *B*  = 0.002, *SE*  = 0.016, *t*  = 1.029, *p*  = 0.311, 95%CI − 0.002–0.048; refugees: *B*  = 2.998, *SE*  = 0.934, *t*  = 3.209, *p*  = 0.021, 95%CI – 1.130–4.883 ); (2) moderate level = 16.0 (control group: B = -0.000, *SE*  = 0.001, *t*  = -0.145, *p=* 0.885, 95%CI -0.003–0.022; refugees: *B*  = 0.988, *SE*  = 0.571, *t*  = 1.730, *p*  = 0.091, 95%CI to -0.165–2.140) and (3) high level = 22.0 (control group: *B*  = -0.004, *SE*  = 0.002, *t*  = -2.227, *p*  = 0.032, 95%CI -0.008 to -0.001; refugees: *B*  = -2.531, *SE*  = 1.758, *t*  = -1.140, *p*  = 0.157, 95%CI − -6.079 to 1.017).

**Table 1:** General characteristics of refugees and controls.

Variable	Refugees (n = 60)	Controls (n = 50)	*p*
Age, years	27.8 ± 6.1	26.1 ± 3.4	0.069
Sex, female	49 (81.7%)	36 (72.0%)	0.232
Education, years	17.3 ± 2.3	13.7 ± 2.9	< 0.001
Somatic disease, yes	32 (53,3%)	26 (2.0%)	0.850
Cigarette smoking, yes	15 (25.0%)	6 (12.0%)	0.090
PQ-16, distress subscale	11.6 ± 9.7	6.8 ± 6.7	0.005
PQ-16, presence subscale	5.8 ± 3.6	3.3 ± 3.1	< 0.001
DACOBS, jumping to conclusions	25.1 ± 5.3	24.6 ± 4.9	0.881
DACOBS, belief inflexibility	18.1 ± 5.6	15.1 ± 3.9	0.001
DACOBS, attention to threat	20.8 ± 5.2	19.0 ± 6.1	0.097
DACOBS, external attribution	14.5 ± 4.5	14.9 ± 3.8	0.554
DACOBS, social cognition problems	18.3 ± 6.1	19.3 ± 5.2	0.549
DACOBS, subjective cognitive problems	21.0 ± 7.0	18.1 ± 6.8	0.035
DACOBS, safety behaviors	11.1 ± 4.9	10.7 ± 4.7	0.615
BDI	13.3 ± 7.6	7.9 ± 7.8	< 0.001
GAD-7	8.5 ± 5.0	6.0 ± 4.7	0.006
IES-R, intrusion	12.8 ± 6.9	10.2 ± 7.1	0.052
IES-R, avoidance	10.1 ± 5.2	7.3 ± 4.9	0.005
IES-R, hyperarousal	11.6 ± 5.8	8.2 ± 5.6	0.003

Data expressed as n (%) or mean ± SD.

Significant p-values after Benjamini-Hochberg correction were marked with bold characters (p ≤ 0.006).

BDI, Beck Depression Inventory; DACOBS, the Davos Assessment of Cognitive Biases Scale; GAD-7, General Anxiety Disorder-7; IES-R, Impact of Events Scale – Revised; PQ-16, the Prodromal Questionnaire-16.

**Table 2:** Biomarkers included in the allostatic load index.

Biomarker	Refugees (n = 60)	Controls (n = 50)	*p*
Cardiovascular markers			
SBP, mmHg	125.5 ± 13.6	125.9 ± 13.7	0.938
DBP, mmHg	80.2 ± 10.5	80.3 ± 7.9	0.890
HR, beats per minute	77.2 ± 12.7	73.3 ± 12.0	0.133
Anthropometric measures			
BMI, kg/m^2^	23.2 ± 4.7	22.4 ± 3.1	0.813
WHR	0.8 ± 0.1	0.8 ± 0.1	0.229
Inflammatory markers			
hsCRP, mg/l	1.7 ± 4.1	1.4 ± 1.7	0.879
Fibrinogen, g/l	2.5 ± 0.5	2.5 ± 0.6	0.919
Albumin, g/dl	4.8 ± 0.2	4.7 ± 0.2	0.021
Il-6, pg/ml	3.1 ± 3.9	2.8 ± 2.2	0.909
Glucose homeostasis			
Glucose, mg/dl	88.9 ± 10.6	85.1 ± 6.5	0.022
Insulin, µU/ml	8.6 ± 4.7	7.8 ± 4.4	0.296
HbA1c, %Hg	5.2 ± 0.2	5.2 ± 0.4	0.413
Lipids			
Cholesterol, mg/dl	180.6 ± 30.3	179.0 ± 27.8	0.774
LDL, mg/dl	100.4 ± 24.4	95.5 ± 27.0	0.316
HDL, mg/dl	63.6 ± 14.4	66.0 ± 14.7	0.389
Triglycerides, mg/dl	84.6 ± 41.4	90.7 ± 36.8	0.120
Steroids			
Cortisol, µg/dl	16.5 ± 23.3	18.6 ± 6.9	< 0.001
DHEA-S, µg/dl	257.0 ± 143.5	285.3 ± 140.1	0.225

Data expressed as mean ± SD.

Significant p-values after Benjamini-Hochberg correction were marked with bold characters (p ≤ 0.006).

BMI, body mass index; DBP, diastolic blood pressure; DHEA-S, dehydroepiandrosterone sulfate; HbA1c, glycated hemoglobin; HDL, high-density lipoproteins, HR, heart rate; hsCRP, high-sensitivity C-reactive protein; IL-6, interleukin-6; LDL, low-density lipoproteins; SBP, SBP, systolic blood pressure; WHR, waist-to-hip ratio.

**Table 3:** Bivariate correlations between the allostatic load index and psychotic-like experiences, psychopathological symptoms and cognitive biases in the whole group of participants.

Variable	Statistics (n = 110)
PQ-16, distress subscale	r = 0.164, p = 0.094
PQ-16, presence subscale	r = 0.186, p = **0.016**
DACOBS, jumping to conclusions	r = 0.033, p = 0.740
DACOBS, belief inflexibility	r = 0.251, p = **0.010**
DACOBS, attention to threat	r = -0.002, p = 0.984
DACOBS, external attribution	r = 0.044, p = 0.653
DACOBS, social cognition problems	r = 0.001, p = 0.992
DACOBS, subjective cognitive problems	r = 0.136, p = 0.163
DACOBS, safety behaviors	r = 0.059, p = 0.547
BDI	r = 0.181, p = 0.064
GAD-7	r = -0.032, p = 0.746
IES-R, intrusion	r = 0.136, p = 0.165
IES-R, avoidance	r = -0.039, p = 0.689
IES-R, hyperarousal	r = 0.037, p = 0.705

Significant p-values were marked with bold characters (p ≤<0.005).

BDI, Beck Depression Inventory; DACOBS, the Davos Assessment of Cognitive Biases Scale; GAD-7, General Anxiety Disorder-7; IES-R, Impact of Events Scale – Revised; PQ-16, the Prodromal Questionnaire-16.

**Table 4:** The results of the moderation analysis.

Variable	B	SE	*T*	*p*	95% CI
Allostatic load index	10.179	3.830	2.657	**0.009**	2.573 to 17.784
Belief inflexibility	0.763	0.265	2.875	**0.005**	0.236 to 1.290
Allostatic load index × Belief inflexibility	-0.592	0.253	-2.341	**0.021**	-1.095 to -0.090
Group	12.650	5.306	2.384	**0.019**	2.114 to 23.186
Allostatic load index × Group	10.613	4.329	2.452	**0.016**	-2.019 to 19.208
Belief inflexibility × Group	-0.529	0.327	-1.616	0.109	-1.178 to 0.121
Allostatic load index × Belief inflexibility × Group	-0.563	0.272	-2.068	**0.018**	-1.104 to 0.022
Age	-0.129	0.071	-1.824	0.071	-0.269 to 0.011
Sex	-0.278	0.071	-0.363	0.717	-1.798 to 1.242
Education years	-0.077	0.130	-0.593	0.554	-0.336 to 0.181
Somatic disease	0.080	0.698	0.115	0.909	-1.307 to 1.467

Significant p-values were marked with bold characters (p ≤<0.005).

## 4 Discussion

In this study, refugees had a significantly higher AL index compared to controls, which is consistent with previous findings among displaced populations (11, 49, 50). Refugees exhibited greater belief inflexibility bias than controls, in line with evidence showing that chronic stress promotes more habitual and inflexible cognitive processing (51). The results showed that higher AL index, greater belief inflexibility, and refugee status were each positively associated with higher levels of PLEs, which has also been found in previous research (52, 53). Similarly, our study found that refugee status was positively associated with PLEs, corresponding with results of another study showing that refugees experience higher levels of PLEs compared to non-refugees (54). Moreover, our findings revealed that, especially among refugees, belief inflexibility significantly moderated the relationship between the AL index and PLEs. Individuals with higher AL index who also showed higher levels of belief inflexibility exhibited lower levels of PLEs. Belief inflexibility showed a dual pattern: it was related to greater PLEs severity at the main-effect level, yet it reduced the strength of the AL–PLEs association under chronic displacement-related stress. Given the relatively modest sample size, these interaction effects should be interpreted cautiously and require replication in larger cohorts. The positive association between belief inflexibility and PLEs severity is consistent with prior evidence indicating that reduced incorporation of disconfirmatory evidence is linked to delusion-proneness and psychosis-spectrum outcomes (36). Experimental paradigms assessing belief flexibility suggest that individuals with schizophrenia-spectrum disorders show diminished revision of strongly held beliefs when confronted with contradictory information (35), and similar alterations have been reported in at-risk and high-schizotypy samples (55). Longitudinal findings further indicate that lower belief flexibility may be associated with persistence of delusional conviction over time (55). Building on this cognitive perspective, meaning-making processes may offer an additional explanatory layer. Meaning making involves integrating traumatic experiences into broader belief systems to restore coherence following adversity (56). In contexts of forced migration, cumulative stress exposure may disrupt global meaning structures and increase reliance on more rigid explanatory frameworks that provide rapid closure (57, 58). Empirical research suggests that difficulties in meaning making are associated with more persistent PTSD symptoms, whereas successful integration of traumatic experiences may predict better adjustment (59). Within cognitive models of psychosis, impaired belief updating and maladaptive meaning attribution may increase the likelihood that ambiguous experiences are interpreted in a delusion-like manner (60, 61). Together, this literature supports the interpretation that belief inflexibility may reflect a cognitive vulnerability marker contributing to the development and persistence of PLEs. However, the moderation findings complicate a simple deficit interpretation. Among refugees with lower belief inflexibility, higher AL was more strongly associated with PLEs severity, whereas this association was attenuated among those with higher belief inflexibility. Under chronic stress exposure, reduced belief updating may limit the extent to which physiological stress dysregulation translates into PLEs. Computational accounts show that psychosis liability is associated with not only under-updating (rigidity) but also over-updating in volatile environments (62, 63). Heightened sensitivity to environmental uncertainty has been linked to unstable belief revision and increased paranoia (64). From this perspective, relative belief inflexibility may reduce excessive updating to ambiguous or stress-related signals, thereby attenuating the association between AL and PLEs under highly uncertain conditions. Evidence from the optimism-biased updating literature provides a complementary framework. A complementary account emerges from the optimism bias refers to the tendency to underweight negative information and maintain overly positive expectations, resembling belief inflexibility in that both involve reduced updating of beliefs in response to undesirable evidence (65). Healthy individuals in save, predictable environment tend to update beliefs more readily in response to desirable rather than undesirable information (66). In contrast, reduced optimism bias and greater incorporation of negative information have been observed in affective disorders (67) and under experimentally induced threat (37). These findings suggest that partial resistance to negative updating may support affective stability under manageable stress. In our sample, higher belief inflexibility may have reduced the degree to which stress-related physiological states indexed by AL were incorporated into maladaptive threat interpretations, thereby weakening the AL–PLEs link. Nonetheless, these interpretations remain tentative and should be tested directly using longitudinal and experimental paradigms.

Taken together, these preliminary findings support a cautious and context-sensitive account of belief inflexibility, consistent with empirical research showing that both reduced and exaggerated belief updating are associated with psychosis risk, depending on environmental volatility and contextual demands (63). This pattern may reflect a trade-off: under sustained adversity, cognitive rigidity may reduce ambiguity and constrain excessive updating, potentially stabilizing experience in the short term, while prolonged reliance on inflexible processing strategies may limit corrective learning and increase longer-term vulnerability. Rather than representing a uniformly maladaptive deficit, belief inflexibility may function as a regulatory cognitive style whose effects depend on stress exposure and environmental volatility. However, given the cross-sectional design, modest sample size, and specific refugee context, the generalizability of these findings remains limited. Larger, longitudinal studies integrating computational measures of belief updating and repeated assessments of AL will be necessary to determine whether belief inflexibility prospectively attenuates the AL–PLEs association or instead reflects a secondary consequence of chronic adversity.

## 5 Limitations and future directions

Our study acknowledges several limitations that should be considered. Firstly, the sample was relatively small and predominantly female, which may restrict generalizability and limit statistical power. Although the sample size was sufficient for main effects, models including multiple covariates and interaction terms may require larger samples for more precise estimation. Therefore, moderation findings, particularly the three-way interaction, should be interpreted with appropriate caution and warrant replication. Secondly the gender imbalance largely reflects wartime migration policies that prevented most Ukrainian men from relocating to Poland. Thirdly, the diverse backgrounds of the refugee group, especially regarding their war-related experiences, may have contributed to variability that was not completely addressed. Additionally, relying on self-reported symptoms could lead to reporting bias, as these subjective measures may not accurately capture the true severity of the symptoms experienced. Additionally, the absence of more objective assessments for PLEs, such as performance-based evaluations, represents a significant gap in psychological research. Moreover, the AL index used in the study is not standardized, making it challenging to generalize our findings in relation to previous research. A limitation of this study is the use of the IES-R, which is based on earlier PTSD models and does not capture the DSM-5 cluster of negative alterations in cognition and mood, potentially limiting the comprehensiveness of PTSD symptom assessment. Finally, our cross-sectional design limits our ability to draw causal conclusions regarding the relationship between biological dysregulations conceptualized through the AL index, PLEs, and cognitive biases. Future research should employ longitudinal and experimental designs to clarify causal relationships among AL, cognitive biases, and PLEs, and to examine whether interventions enhancing adaptive cognitive control and stress regulation could mitigate psychosis risk among refugees. Integrating AL assessment with neurofunctional and neuroendocrine measures may further elucidate the mechanisms linking stress physiology, cognition, and psychosis vulnerability in this population.

## Data Availability

The raw data supporting the conclusions of this article will be made available by the authors, without undue reservation.
